# Tissue Regeneration and Biomineralization in Sea Urchins: Role of Notch Signaling and Presence of Stem Cell Markers

**DOI:** 10.1371/journal.pone.0133860

**Published:** 2015-08-12

**Authors:** Helena C. Reinardy, Chloe E. Emerson, Jason M. Manley, Andrea G. Bodnar

**Affiliations:** Molecular Discovery Laboratory, Bermuda Institute of Ocean Sciences, St. George’s GE 01, Bermuda; Chang Gung University, TAIWAN

## Abstract

Echinoderms represent a phylum with exceptional regenerative capabilities that can reconstruct both external appendages and internal organs. Mechanistic understanding of the cellular pathways involved in regeneration in these animals has been hampered by the limited genomic tools and limited ability to manipulate regenerative processes. We present a functional assay to investigate mechanisms of tissue regeneration and biomineralization by measuring the regrowth of amputated tube feet (sensory and motor appendages) and spines in the sea urchin, *Lytechinus variegatus*. The ability to manipulate regeneration was demonstrated by concentration-dependent inhibition of regrowth of spines and tube feet by treatment with the mitotic inhibitor, vincristine. Treatment with the gamma-secretase inhibitor DAPT resulted in a concentration-dependent inhibition of regrowth, indicating that both tube feet and spine regeneration require functional Notch signaling. Stem cell markers (Piwi and Vasa) were expressed in tube feet and spine tissue, and Vasa-positive cells were localized throughout the epidermis of tube feet by immunohistochemistry, suggesting the existence of multipotent progenitor cells in these highly regenerative appendages. The presence of Vasa protein in other somatic tissues (e.g. esophagus, radial nerve, and a sub-population of coelomocytes) suggests that multipotent cells are present throughout adult sea urchins and may contribute to normal homeostasis in addition to regeneration. Mechanistic insight into the cellular pathways governing the tremendous regenerative capacity of echinoderms may reveal processes that can be modulated for regenerative therapies, shed light on the evolution of regeneration, and enable the ability to predict how these processes will respond to changing environmental conditions.

## Introduction

The ability to renew or repair damaged tissue varies widely among organs and organisms. While some organisms have very limited ability to regenerate tissues, others can replace entire organs or appendages repeatedly throughout life [1]. Members of the phylum Echinodermata have tremendous regenerative capabilities and regeneration is common in all Echinoderm classes: crinoids (feather stars), asteroids (sea stars), ophiuroids (brittle stars), echinoids (sea urchins), and holothuroids (sea cucumbers). Regeneration is employed to reconstruct external appendages that are subject to predation or amputation, and internal organs following evisceration [2]. In addition, some asteroids, ophiuroids, and holothuroids can undergo fission whereby adults can divide into two or more parts with subsequent regenerative development of complete individuals from each resultant piece [2]. Regeneration in echinoids is less well studied than other Echinoderm classes; however, they offer tractable models for molecular and cellular research on regeneration. Sea urchins readily regenerate external appendages (e.g. spines, pedicellariae, tube feet), providing an opportunity to investigate distinct regenerative processes. Because sea urchins are a well-established model of developmental biology, there are many molecular and cellular tools available including the complete genome of *Strongylocentrotus purpuratus* and extensive DNA sequence for other species, including *Lytechinus variegatus* [3,4] (www.echinobase.org). These tools enable genome-wide profiling of gene and protein expression at different stages of regeneration or in response to agents that perturb particular cellular pathways. In addition, the gene regulatory networks that control sea urchin development are well characterized and provide a framework to determine the degree to which regeneration recapitulates developmental pathways [5–7]. As with all echinoderms, sea urchins are non-chordate deuterostomes that share a close phylogenetic relationship with humans and therefore may produce findings that can be extended to human regenerative therapies. Mechanistic insight into the cellular pathways governing the tremendous regenerative capacity of echinoderms may also shed light on the evolution of regeneration and enable the ability to predict how these processes will respond to changing environmental conditions.

The unique physical properties of sea urchin spines are well studied and have been shown to consist of a large single crystal of magnesium-containing calcite [8,9]. Spine biomineralization is driven by skeletogenic cells (sclerocytes) located in the dermis that covers the surface of the sea urchin skeleton (an endoskeleton). Spine regeneration initially involves a wound-healing process where the epidermis is reconstituted around the broken spine. Calcification then takes place in a syncytium formed by the sclerocytes [8,9]. The cellular and molecular pathways involved in spine regeneration are not characterized, but the gene regulatory networks and signaling pathways associated with skeletogenesis in sea urchin embryos and the juvenile rudiment are well understood [10–12].

Tube feet are fleshy extensions of the water vascular system that protrude through the sea urchin shell and play a role in locomotion, respiration, and sensory perception. There are about 1500 tube feet per sea urchin, each comprised of several well-defined layers: an outer epidermis, a basiepidermal nerve plexus, a connective tissue layer and a longitudinal muscle layer lined with ciliated epithelium facing the inner water vascular lumen [13–15]. A disc at the distal end of each tube foot is used for adhesion and also receives sensory input which is transduced to the radial nerve which lies just inside the test [14]. Tube feet provide a convenient model for regeneration particularly relevant to nerve and muscle tissue, however there are no studies describing regeneration of these appendages.

Regeneration in echinoderms can employ both morphallactic and epimorphic processes involving differentiated and dedifferentiated cells [2,16]. It has been suggested that pluripotent cells are also involved, however the existence of stem cells in somatic tissues of echinoderms has not been demonstrated [2,16]. Genome-wide profiling of expression during regenerative processes and loss- or gain-of-function studies are not yet possible in sea stars, sea cucumbers, brittle stars, or feather stars due to lack of genomic resources. Nevertheless, gene expression studies using these organisms have implicated many of the same cellular pathways employed in regenerative processes of other animals such as the bone morphogenic protein (BMP), Hox, and Wnt pathways [17–20]. In many biological systems, Notch signaling plays a role in embryonic development, homeostasis of adult tissues, and stem cell function [21]. Notch receptors are activated when they interact with membrane bound ligands of the Delta or Serrate/Jagged families on adjacent cells. This interaction leads to proteolytic cleavage of the Notch receptor by γ-secretase to release the Notch intracellular domain (NICD), which translocates to the nucleus. In the nucleus, NICD interacts with transcriptional regulators to modify the expression of target genes such as transcription factors of the Hes and Hey families [21]. Notch signaling has been shown to be required for regeneration of *Xenopus* larval tails [22], zebrafish fins [23], and mammalian skeletal muscle [24]. Although Notch signaling has been shown to be involved in endomesoderm segregation and specification of the non-skeletogenic mesoderm in sea urchin embryos [25], there are no reports of the involvement of the Notch signaling pathway in regeneration in adult echinoderms.

Progress in understanding the underlying mechanisms of regeneration in echinoderms depends on the continued development of genetic tools and functional approaches to manipulate regenerative processes in these animals. In the current study we have developed an assay to measure regenerating tube feet and spines and have successfully manipulated these processes through intracoelomic injections of pharmacological agents. The results suggest that the Notch signaling pathway is involved in both tube feet and spine regeneration. Given the important role that Notch signaling plays in stem or progenitor cell function, we examined the expression of the stem cell markers, Piwi and Vasa, in tube feet and the tissue associated with spines. Piwi and Vasa were chosen for their role in a variety of multipotent stem cell types across many animal phyla. Vasa is a DEAD-box RNA helicase that acts as a translation regulator, but it has also been shown to play a role in pre-mRNA splicing, ribosome biogenesis, and nuclear export [26]. Piwi belongs to the highly conserved Piwi/Argonaute family that binds to specific micro-RNAs called piRNAs, which act in transposon silencing and regulation of transcriptional activity [27]. Vasa and Piwi play a role in germline development and maintenance in *Drosophila melanogaster*, *Caenorhabditis elegans*, and mammals (mice and humans). However, in some animals (e.g. cnidarians, planarians, tunicates), expression of Vasa and Piwi are not restricted to the germline but are found in multipotent stem cells that are capable of producing both somatic and germline derivatives [26,28]. The presence of Vasa and Piwi in the tissues of adult sea urchins would suggest the existence of multipotent progenitor cells that may underlie their high regenerative capacity.

## Materials and Methods

### Collection and Maintenance of sea urchins

*L*. *variegatus* sea urchins were collected in Bermuda from Helena’s Bay (32°22'N and 64°42'W), Mangrove Bay (32°22'N and 64°42’W), and Harrington Sound (32°19'N and 64°43'W). Animals were maintained in flow-through aquaria and were fed a constant supply of macroalgae and sea grass (*Thalassia testudinum*), augmented with shredded lettuce. The study organisms are invertebrates and as such no restrictions apply to their handling as experimental organisms, however all animal collections, maintenance, and experimental protocols complied with the Collecting and Experimental Ethics Policy (CEEP) of the Bermuda Institute of Ocean Sciences. All collections were considered Limited Impact Research except the collections of *L*. *variegatus* from Mangrove Bay which were conducted under collection permits 140409 and 140803 from the Government of Bermuda, Department of Environmental Protection. *T*. *testudinum* was collected under License no. 14-09-01-12 from the Government of Bermuda Department of Conservation Services.

### Sea urchin regeneration assay

A single strip of tube feet and spines was removed from one ambulacral section by cutting along the test with dissecting scissors while the sea urchin was underwater. A secondary finer cut ensured that all tube feet and spines were trimmed as close to the test as possible. After amputations, sea urchins were left to recover overnight before start of treatments. Spines and tube feet were measured weekly over four weeks (1, 8, 15, 22, and 29 days post amputation, dpa). Six uncut spines were measured at the start (1 dpa) and end (29 dpa) of the experiment using electronic calipers, and measurements were averaged to give the mean full spine length (n = 12). Regrowing spines (n = 6) were measured with electronic calipers at each sampling time, and regeneration for each animal was estimated by mean length of regrowing spines, as a percentage of mean full-length spines from the same animal. Tube feet were measured from images photographed at each sampling time. Sea urchins were placed in a shallow tub filled with seawater and a ruler was positioned vertically, adjacent to the cut section. Sea urchins were left to relax and extend their tube feet before being photographed using an underwater camera (Panasonic Lumix DMC-TS5). Images showing well-extended tube feet were selected, and a single image was analysed per animal. Selected photographs were uploaded into FIJI [(Fiji Is Just) ImageJ, ImageJ1.49b, [29]], and uncut and regrowing tube feet were measured using the freehand drawing tool with the scale set according to the ruler in the image. A minimum of 10 full-length and 10 regrowing tube feet along the length of the cut section were averaged for each animal at each time point, and regeneration was estimated by mean length of regrowing tube feet, as a percentage of mean full-length tube feet within the same image. Regeneration rate was calculated by linear regression using Statgraphics X64 (Statgraphics Centurion XV1.11, StatPoint Technologies, USA).

### Pharmacological Treatments

Pharmacological treatments were administered via injection into the body cavity through the peristomial membrane thrice weekly (starting 1 dpa) for a total of 13 injections over the course of the 4-week experiments. Vincristine sulfate (Sigma-Aldrich, V8388) was diluted into calcium- magnesium-free artificial seawater (460 mM NaCl, 10 mM KCl, 7 mM Na_2_SO_4_, 2.4 mM NaHCO_3_, pH 7.4) for individual delivery of 0, 0.2, or 0.6 μg vincristine per gram body weight, in 250 μl injections. N-[N-(3,5-Difluorophenacetyl)-L-alanyl]-S-phenylglycine t-butyl ester (DAPT, Sigma-Aldrich, D5942) working stocks were prepared in dimethyl sulfoxide for individual delivery of 0, 1, 3, or 9 μg DAPT per gram body weight, in 80 μl injections. Twenty-four hours after the final injection with DAPT, tube feet were cut and stored in RNA*later* solution (Qiagen, CA) at -80°C prior to gene expression analysis. Statistical tests were conducted with Statgraphics X64. Regeneration of spines and tube feet was calculated to be the length of regrowth as a percentage of animal-matched full lengths. Percentage data was arcsine transformed, and overall effect of time and concentration on regeneration was tested by general linear model (GLM); within time point concentration differences were tested by one-way ANOVA with *post-hoc* multiple range tests (MRT). Rates of regeneration were tested by linear regression.

### Gene expression analyses

Total RNA was extracted from tube feet and spines using the Trizol reagent (Life Technologies, CA) followed by the RNA clean-up protocol (RNeasy mini Kit, Qiagen) with a 15-min DNase digestion step (Qiagen). cDNA was synthesized (High-Capacity cDNA Reverse Transcription Kit, Applied Biosystems, CA) and analysed by quantitative reverse-transcription PCR (qRT-PCR, ABI 7300 Real Time-PCR) using the SYBR Green detection system (Applied Biosystems). PCR conditions included initial denaturation (90°C, 10 min.) and 40 cycles (95°C for 15 sec. and 60°C for 1 min.) followed by dissociation curve analysis. Primers were designed using Primer Express software (version 3.0, Applied Biosystems) using sequences of *L*. *variegatus* target genes identified in the echinoderm genome database (www.echinobase.org) (S1 Table). Primer concentrations were optimized and PCR efficiency was calculated for each primer pair (*E* = 10^(-1/slope)^ [30]. Control genes (*cyclophilin7*, *rpl8*, *profilin*, and *ubiquitin*) were selected from a panel of genes that are consistently expressed across different sea urchin tissues and size/age categories [31]. Control genes were analyzed for stability (Biogazelle, qbase+ 2.6.1 [32]) and tested for effect of treatment (one-way ANOVA, p > 0.05). Differential expression of genes of interest was determined using the delta-delta-Ct method normalized to the three most stably expressed control genes (geometric mean) and relative to untreated control animals [30]. Treatment effects on relative fold change in gene expression were tested by one-way ANOVA where the data complied with normality and Kruskal-Wallis for non-parametric data.

### Immunohistochemistry/Immunocytochemistry

Tissues and cells were fixed in 4% paraformaldehyde in PBS. Tissues were embedded in paraffin, sectioned (5 μm), and mounted on slides. Slides were deparaffinized with toluene, rehydrated, and unmasked with 10 mM sodium citrate solution at 95°C for 20 minutes. Tissues and cells were permeabilized in methanol at -20°C for 5 minutes. Blocking was conducted for 2 hours in 4% bovine serum albumin in PBST (PBS with 0.1% Tween 20), followed by an overnight incubation at 4°C with 1 μg/ml anti-vasa antibody (Developmental Studies Hybridoma Bank). Negative controls lacking the primary antibody were also conducted. Samples were washed with PBST and incubated for 2 hours with DyLight 488 as a secondary antibody (1:1000 dilution, 112-486-075, Jackson Immunoresearch). After washing with PBST, slides were mounted with Citifluor containing 4',6-diamidino-2-phenylindole (DAPI, 1.67 μg/ml) and analysed on an Olympus AX70 epiflourescent microscope. Images were captured with a Retiga EXi Digital camera (Qimaging, BC, Canada) and recorded using Image Pro Plus version 7.0 software (Media Cybernetics, MD).

## Results

### Sea urchin regeneration assay

**Fig 1:**
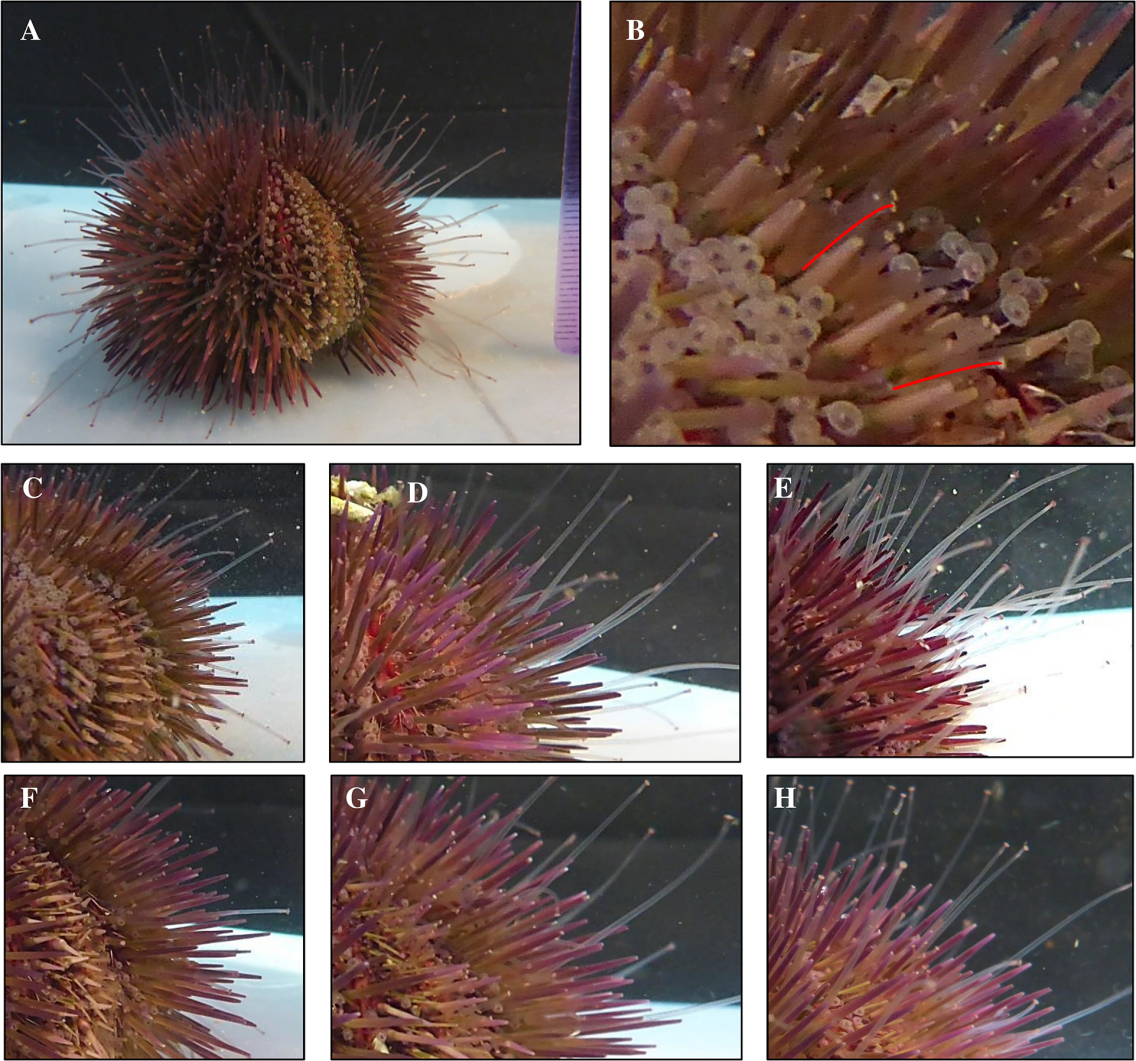
Sea urchin regeneration assay. A single strip of tube feet and adjacent spines from one ambulacral section were cut at the base (A, 1 day post amputation, dpa). Regrowth of spines was measured directly with calipers, and tube feet length was measured by image analysis of underwater photographs (B, 8 dpa), with the scale set from a ruler placed adjacent to cut section (visible on right, panel A). Panels C–E indicate regrowth in a control animal after 8, 15, and 22 dpa, respectively. Panels F–H indicate regrowth in an animal treated with 3 μg/g DAPT after 8, 15, and 22 dpa, respectively.

Regeneration in sea urchin tube feet and spines was quantified, each week for 4 weeks after amputation, by direct measurement of spines and image analysis of tube feet (Fig 1). Spine and tube feet regeneration were expressed as a percentage of full-length (not amputated) spines and tube feet from each individual. Image analysis of tube feet posed several technical challenges due to the varying extension and direction of the tube feet. These problems were overcome by ensuring that each animal relaxed and fully-extended its tube feet prior to imaging, selecting tube feet that were extended in a direction perpendicular to the camera’s line of sight, and averaging the results from a minimum of 10 regrowing tube feet along the amputated section and 10 full-length tube feet for each individual (for spines, 6 measurements were averaged). Data sets were verified using blinded tests of multiple images of individual animals. No significant differences were found between results from blinded and not-blinded images from the same data set and the percent regeneration was consistent using different images of the same animal.

**Fig 2:**
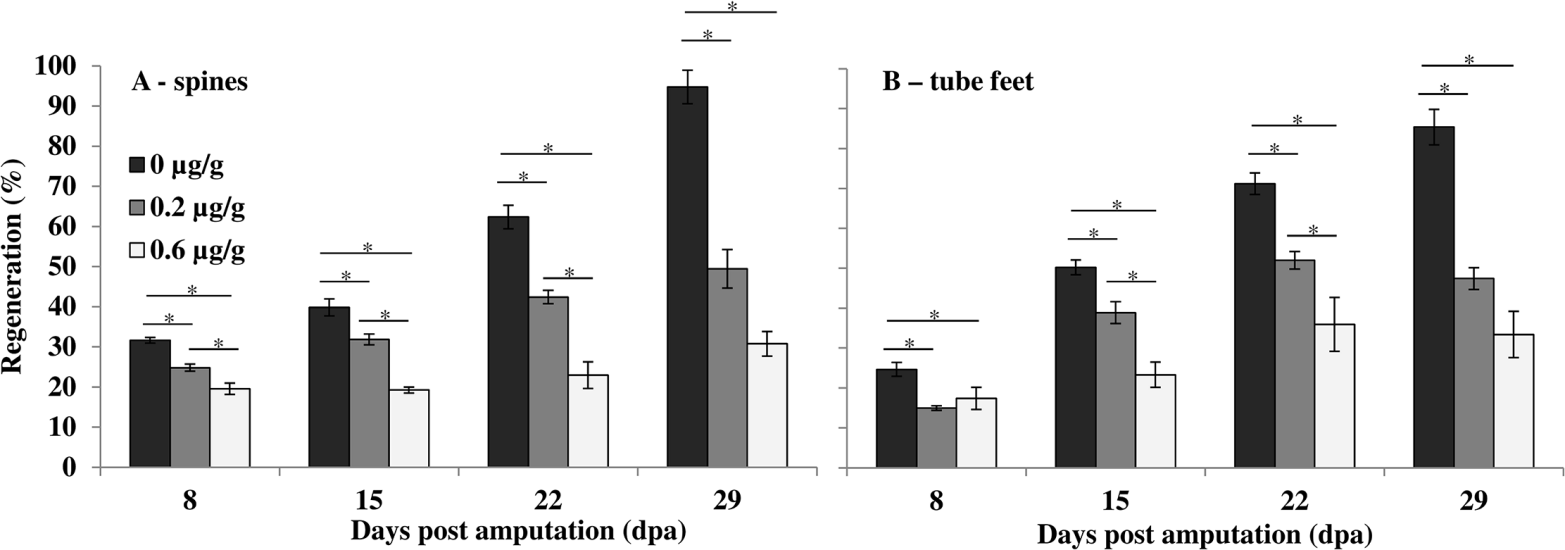
Spine and tube feet regeneration following treatment with the mitotic inhibitor, vincristine. Regeneration (% of full-length, uncut appendages) in spines (A) and tube feet (B) after treatment with 0 (black bars), 0.2 μg/g (grey bars), and 0.6 μg/g (white bars) vincristine. Data are means ± s.e.m., n = 4 individuals (except 29 dpa, n = 3, 0 μg/g, and n = 2, 0.6 μg/g due to mortality prior to measurement). *Significant reduction in regeneration with concentration of vincristine (arcsine-transformed, One-way ANOVA, *post-hoc* MRT, p<0.05).

The ability to modulate regeneration was tested by treatment with the mitotic inhibitor, vincristine. An initial qualitative trial experiment resulted in inhibition of regeneration with no visible regrowth of spines or tube feet after two weeks of treatment with vincristine (1 μg/g body weight). In a second experiment using 0.2 and 0.6 μg/g there was a significant effect of time and concentration on the rates of regrowth (GLM, p < 0.05), and a significant concentration-dependent inhibition of regeneration of both spines and tube feet at each time point throughout the experiment (8–29 days post amputation, dpa; p < 0.05, arcsine transformed, one-way ANOVA, Fig 2). Control animals regrew tube feet on average 0.46 ± 0.09 mm/day (3.10 ± 0.28% per day, R^2^ = 95.6, p < 0.05) and spines on average 0.38 ± 0.02 mm/day (3.18 ± 0.19% per day, R^2^ = 88.5, p < 0.05). The highest treatment group regrew their tube feet on average 0.06 ± 0.05 mm/day (0.49 ± 0.30% per day, R^2^ = 58.7, p < 0.05) and spines on average 0.05 ± 0.03 mm/day (0.41 ± 0.28% per day, R^2^ = 56.7, p < 0.05). At 29 dpa the control animals showed 85 ± 4% and 95±4% regrowth of tube feet and spines, respectively, while the group treated with 0.6 μg/g vincristine exhibited significantly reduced regrowth of only 33 ± 6% and 31 ± 3% for tube feet and spines, respectively (p < 0.05, arcsine transformed, one-way ANOVA, Fig 2). It is likely that initial measurements of regrowth at 8 dpa includes both a single week’s regenerative growth in addition to residual uncut spine and tube foot base; therefore rates of regeneration were calculated from 8 dpa. Spine and tube feet measurements for each animal at each time point are shown in S2 Table.

### Role of Notch signaling

**Fig 3:**
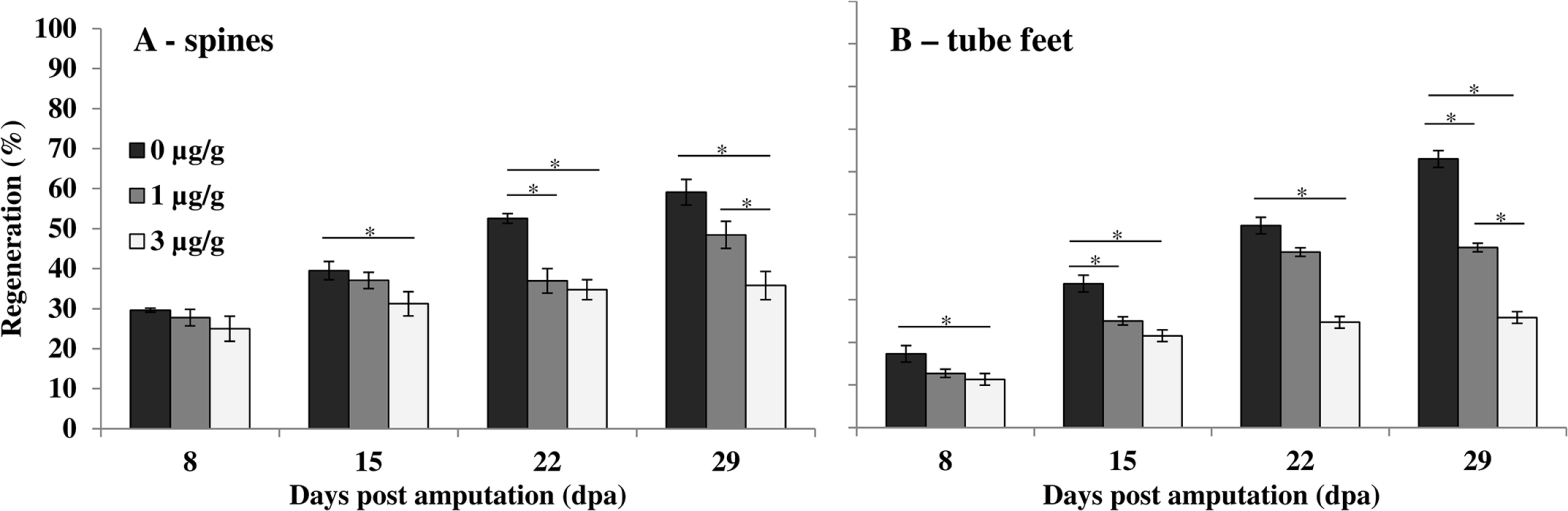
Spine and tube feet regeneration following treatment with DAPT. Regeneration (% of full-length, uncut appendages) in spines (A) and tube feet (B) after treatment with 0 (black bars), 1 μg/g (grey bars), and 3 μg/g (white bars) DAPT. Data are means ± s.e.m., n = 4 individuals (except tube feet from 0 μg/g, 29 dpa, n = 3 due to mortality prior to measurement). *Significant reduction in regeneration with concentration of DAPT (arcsine-transformed, One-way ANOVA, *post-hoc* MRT, p<0.05).

**Fig 4:**
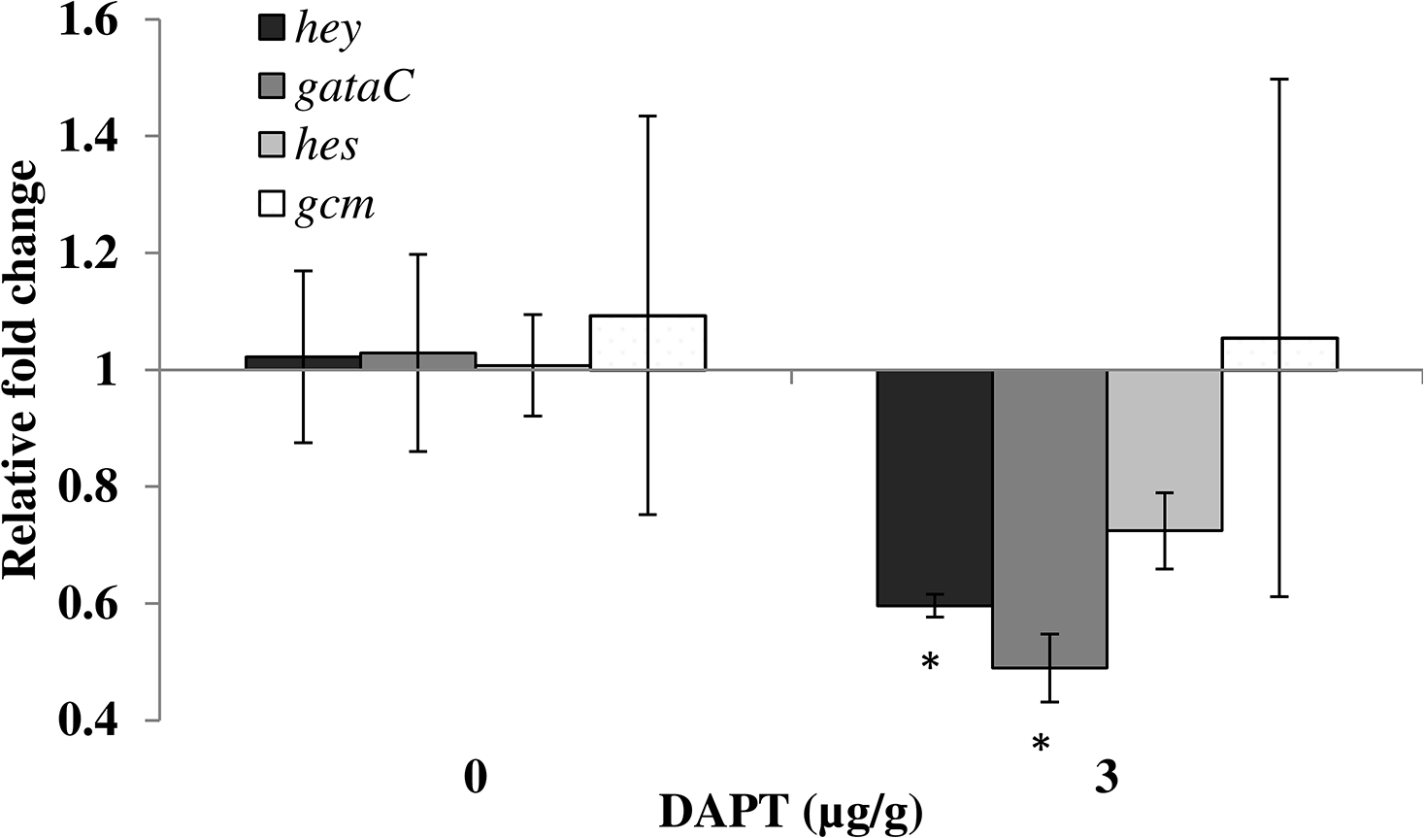
Inhibition of Notch signaling in regenerating sea urchins treated with DAPT. Tube feet sampled 24 hours after final treatment with DAPT, 29 days post amputation. Gene expression (qRT-PCR) of selected Notch target genes (*hey*, *gataC*, *hes*, *gcm*), compared with the geometric mean of three most stable control genes (*cyclophilin7*, *rpl8*, *profilin*), data are geomeans ± s.e.m., n = 3 animals, *significant down-regulation (p < 0.05).

Three independent experiments were conducted that showed inhibition of spine and tube feet regeneration with treatments of the Notch signaling inhibitor, DAPT. An initial experiment conducted over 15 days found significant inhibition of tube feet regrowth at 1 and 3 μg/g and significant inhibition of spine regrowth at 3 μg/g (spine and tube feet measurements are listed in S3 Table). This experiment was repeated with regeneration followed over 29 days. Overall, there was a significant effect of time and concentration on regeneration (arcsine transformed, GLM, p < 0.05). Significant reduction in regeneration at the 3 μg/g treatment level was seen after 8 days regrowth in tube feet and after 15 days of regrowth in spines (Fig 3). The rate of tube feet regrowth declined from control levels of 0.97 ± 0.12 mm/day (2.59 ± 0.06% per day, R^2^ = 83.3, p < 0.05) to 0.38 ± 0.08 mm/day (0.72 ± 0.17% per day, R^2^ = 58.7, p < 0.05) in the highest treatment group, and spine regrowth declined from control levels of 0.14 ± 0.01 mm/day (1.53 ± 0.15% per day, R^2^ = 90.4, p < 0.05) to 0.05 ± 0.01 mm/day (0.54 ± 0.14% per day, R^2^ = 36.0, p < 0.05) in the highest treatment group. There was a significant concentration-dependent inhibition of regeneration with a 2.4-fold and 1.7-fold reduction in tube feet and spine regrowth, respectively, in animals treated with 3 μg/g DAPT after 29 dpa (p < 0.05, arcsine transformed, one-way ANOVA). Spine and tube feet measurements for each animal at each time point are shown in S4 Table. The experiment was repeated for a third time, conducted over 29 days with three concentrations of DAPT (1, 3, and 9 μg/g). The pattern of significant concentration-dependent inhibition of regeneration in both spines and tube feet was maintained for 1 and 3μg/g, but the animals treated with 9μg/g DAPT died at 9 dpa (spine and tube feet measurements are shown in S5 Table). There was significant down-regulation of expression of Notch target genes *hey* (0.60 ± 0.02 relative fold change, p < 0.05, one-way ANOVA), *gataC* (0.49 ± 0.06 relative fold change, p < 0.05, Kruskal-Wallis), and *hes* (0.72 ± 0.07 relative fold change, p = 0.05, Kruskal-Wallis), in tube feet sampled 24 hours after final treatment of 3 μg/g (29 dpa). There was no clear down-regulation and high inter-individual variability of *gcm* in the same samples (Fig 4).

### Expression of stem cell markers

**Fig 5:**
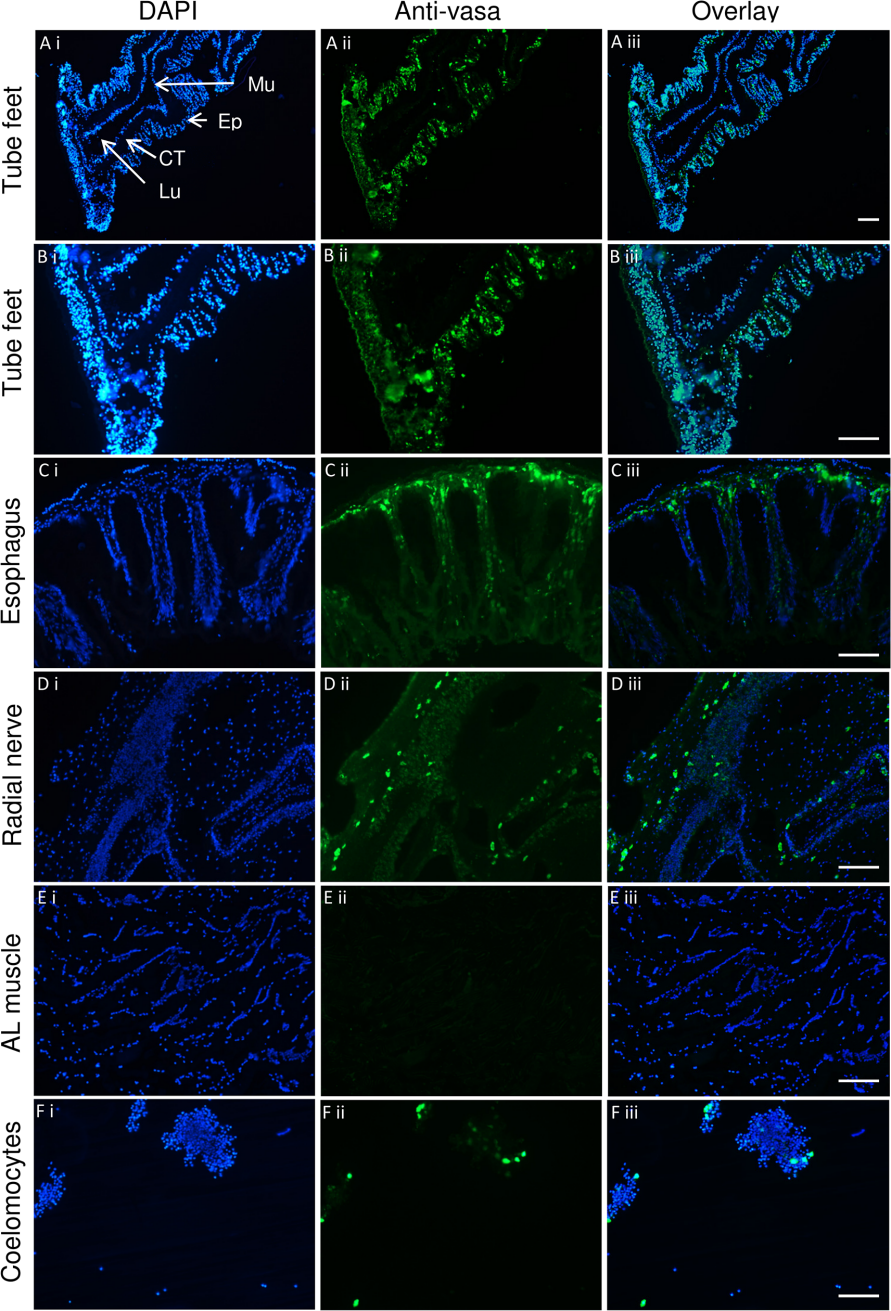
Stem cell marker, Vasa, in sea urchin adult tissues. Immunohistochemistry of tube foot [A and B (detail); Mu = muscle, Ep = epidermis, CT = connective tissue, Lu = lumen], esophagus (C), radial nerve (D), Aristotle’s lantern muscle (E), and coelomocytes (F) stained with DAPT (i), antibody to vasa visualized with DyLight 488 secondary antibody (ii), vasa/DAPI image overlay (iii). Representative images from n = 6 (tube feet) or n = 4 (other tissues) individuals. Scale bar is 100 μm.

The expression of stem cell marker genes, *piwi* and *vasa*, was demonstrated in mRNA isolated from untreated, homeostatic tube feet and spines (Table 1). mRNA levels, estimated by qRT-PCR cycle threshold (Ct), of *vasa* were within the Ct range of control genes (*ubiquitin*, *cyclophilin7*, *rpl8*, *profilin*), and *piwi* mRNA was well within detection range. Immunohistochemical analysis of tube feet demonstrated the presence of the Vasa protein throughout the epidermis of the stalk and disc but absence in the muscle and connective tissue layers that line the lumen (Fig 5). Immunohistochemical analysis of other tissues showed Vasa-positive cells in esophagus and radial nerve, and in a sub-population of coelomocytes (circulating immune cells), but little staining in muscle from Aristotle’s lantern (Fig 5). The anti-vasa antibody used in this study was produced against *D*. *melanogaster* Vasa, but has been previously shown to detect sea urchin Vasa in the small micromeres of *S*. *purpuratus* embryos [33]. The antibody was raised to amino acids 16–433 of *D*. *melanogaster* Vasa and an alignment of this region with *L*. *variegatus* Vasa shows good sequence conservation (37.6% identity, 51.6% similarity) across the entire region and stronger conservation for amino acids 202–433 (46.6% identity and 66.8% similarity) (S1 Fig).

**Table 1:** Gene expression of stem cell markers in sea urchin tube feet and spines. Data are means ± s.e.m., n = 6 individuals.

Gene	qRT-PCR cycle threshold (Ct)	
	Tube feet	Spines
*vasa*	22.0 ± 0.4	25.2 ± 0.1
*piwi*	27.4 ± 0.4	24.5 ± 0.2
*ubiquitin*	17.8 ± 0.4	17.4 ± 0.1
*cyclophilin7*	20.3 ± 0.4	19.7 ± 0.1
*rpl8*	20.6 ± 0.5	19.7 ± 0.1
*profilin*	25.5 ± 0.5	22.5 ± 0.2

## Discussion

The lack of functional studies to investigate mechanisms of regeneration in echinoderms prompted us to devise an assay to both measure and manipulate regenerative processes in sea urchins. This assay enables investigation of two distinct regenerative processes: soft tissues including nerve and muscle associated with tube feet, and spine biomineralization controlled by the dermis and epidermis. This novel and straightforward assay is a promising method to investigate mechanisms of regeneration due to the ability to measure regrowing and uncut appendages from the same animals and the ease of administration of agents into the coelomic cavity to disrupt cellular processes. Vincristine, a well characterized mitotic inhibitor, has been shown to inhibit cell division in developing sea urchin embryos [34] and was therefore selected to demonstrate that regeneration of tube feet and spines could be modulated by pharmacological agents injected into the coelomic cavity. Vincristine treatment resulted in a dose-dependent inhibition of both spine and tube feet regeneration. Although we do not have direct evidence for vincristine-induced mitotic arrest, such as reduced BrdU incorporation, this is the most likely possibility for the observed result. *L*. *variegatus* is well suited for these studies due to the relatively fast rate of growth with detectable treatment effects within one week and significant regrowth of amputated appendages within one month. The rapid rate of regeneration is consistent with regrowth of brittle star arms (0.14–0.4 mm/day) [35], and eviscerated internal organs in the sea cucumber *Holothuria glaberrima* which can complete regrowth within 3–5 weeks [36,37]. There was some variation in the overall rate of regrowth of spines and tube feet between different experiments which may be related to differences in sea water conditions such as seasonal temperature variations. Environmental conditions can influence rates of regeneration; slower regrowth of amputated brittle star arms is correlated with colder water [38] and lower pH [39]. The assay used in the present study would be useful to investigate the effects of changing environmental conditions on regeneration in adult sea urchins. The regeneration assay generated reproducible results even with small sample sizes (n = 4 for each treatment group), however the sensitivity may be improved with larger sample numbers to account for inter-individual variability and to detect subtle differences between groups. The availability of genomic information for sea urchins (www.echinobase.org) facilitates genome-wide profiling of gene and protein expression at different stages of regeneration or in response to agents that perturb particular cellular pathways. It also enables the potential for genetic knock-down experiments using systemic delivery technologies such as vivo-morpholinos [40].

This is the first study to investigate mechanisms underlying spine and tube feet regeneration in adult sea urchins. The Notch signaling pathway has been associated with endomesoderm segregation and mesoderm specification in sea urchin embryos [25], and in tissue regeneration of other organisms (e.g. frogs, zebrafish, mice, hydra) where it controls the balance between proliferation and differentiation of precursor cells [21,24,41]. In vertebrates, the outcome of Notch signaling during regeneration is highly context dependent; in some tissues (e.g. muscle and nerve) it appears to maintain progenitor cell status whereas in others (e.g. epidermis) it promotes differentiation [21,42]. In hydra, Notch signaling is required for head regeneration where it maintains the hypostomal (head organizer) precursor cells and suppresses the tentacle cell fate [41]. Chemical inhibition of Notch signaling with DAPT resulted in inhibition of regrowth of amputated tube feet and spine of sea urchins suggesting that Notch is also essential for these distinct regenerative processes. Down regulation of Notch signaling was confirmed by the decreased expression of target genes (*hey*, *gataC*, and *hes*) in tube feet measured 24 hours after the final injection. *Gcm* is a known target of Notch signaling necessary in early sea urchin development however, after its initial activation, sustained expression appears to be independent of Notch [25], which may explain the lack of change observed in this study. Future studies could investigate the effect of inhibiting Notch on other cellular pathways and work toward building a gene regulatory network that details how Notch interacts with other signaling pathways (e.g. BMP, Wnt, Hox) to regulate regeneration. It will be important to determine whether Notch signaling is only activated in response to injury. However, decrease in target gene expression in non-regenerating tube feet in response to DAPT treatment indicates that Notch may be involved in normal tissue homeostasis in these animals. Sea urchins exhibit indeterminate growth in addition to high regenerative capabilities and sustained Notch signaling may be important for both these properties. DAPT, a dipeptide inhibitor of γ-secretase, is widely used to inhibit Notch signaling; however, it is important to note that γ-secretase has other targets in addition to the Notch proteins [43] and more selective inhibitors would be helpful to verify Notch involvement in tissue regeneration in sea urchins.

It has been suggested that stem cells underlie the high regenerative potential of echinoderms [2,44], but stem cells have not yet been identified in adult somatic tissues. In this study, we detected the expression of two stem cell markers (Vasa and Piwi) in tissue associated with spines and tube feet. Immunohistochemical localization of the Vasa protein in tube feet showed staining in the epidermis of the stalk and distal disc, but not in the muscle or connective tissue. Although originally characterized as a germline marker, Vasa has been shown to be expressed in multipotent stem cells that give rise to both somatic and germline derivatives across many animal phyla (e.g. cnidarians, planarians, tunicates) [26,28]. Like echinoderms, these animals possess high regenerative capabilities and it is tempting to speculate that Vasa-positive cells, located in the epidermal tissue along the length of tube feet, may be multipotent cells that underlie their high regenerative potential. The presence of Vasa protein in other somatic tissues (e.g. esophagus, radial nerve, and a sub-population of coelomocytes) indicates that it is not restricted to the highly regenerating tube feet and spines and may play a more general role in tissue homeostasis sea urchins. The absence of Vasa in muscle (in tube feet and Aristotle’s lantern) indicates that not all tissues have resident multipotent cells. It has been previously suggested that circulating stem cells are involved in regenerative processes in echinoderms [2,44] and the identification of a sub-population of Vasa-positive coelomocytes supports this assertion. Although the presence of Vasa and Piwi is strongly suggestive of stem cell properties, definitive proof requires demonstration that these cells are undifferentiated and have the capability to differentiate into different cell types. If so, it would be interesting to ascertain their origin and fate through lineage tracing experiments. In sea urchin embryos, Vasa accumulates selectively in the small micromeres; multipotent cells that give rise to the somatic and primordial germ cells of the adult rudiment [45]. Following metamorphosis, Vasa is expressed in the germ cells of the developing juvenile gonads [45], but the presence of Vasa in adult somatic tissues suggests that it is reactivated in later life perhaps to support homeostatic and regenerative processes.

This study presents a functional assay to measure and manipulate regenerative processes using sea urchins and provides an opportunity to investigate mechanisms underlying the tremendous regenerative capacity of these echinoderms. We have shown that Notch signaling is essential for both tube feet and spine regeneration and have localized the expression of stem cell markers to these tissues implying the existence of multipotent progenitor cells. This opens the door for future studies investigating the activity of these putative stem cells in normal tissue homeostasis and tissue regeneration in sea urchins. Mechanistic insight into the cellular pathways governing regeneration across diverse organisms will offer a deeper understanding of the evolution of regeneration and inform on why regenerative capabilities vary so widely between different organisms. It has been suggested that the tremendous regenerative capabilities of echinoderms underlies their evolutionary success [2], therefore understanding how regenerative processes respond to changing environmental conditions is paramount to predicting the future vulnerability or success of these keystone marine animals.

## Supporting Information

S1 Fig EMBOSS Needle sequence alignment of *Drosophila melanogaster* vasa, amino acids 16–433 (GenBank: AAF53438), with *Lytechinus variegatus* vasa (GenBank ACM80368). (DOCX)

S1 Table Gene-specific primers for *L*. *variegatus* for qRT-PCR analyses. Annotated sequences for *S*. *purpuratus* were used to identify homologous sequences in the *L*. *variegatus* genome for primer design (www.echinobase.org).
(DOCX)

S2 Table Appendage (spines and tube feet) lengths from sea urchins treated with vincristine and following regeneration over 29 days post amputation (dpa). Appendage length data are means, ± s.e.m., n = 12 (full length spines), n = 6 (regenerating spines), n = 10 (tube feet, TF).
(DOCX)

S3 Table Appendage (spines and tube feet) lengths from sea urchins treated with DAPT and following regeneration over 15 days post amputation (dpa) (initial experiment). Appendage length data are means, ± s.e.m., n = 18 (full length spines), n = 6 (cut spines), n = 10–30 (tube feet, TF).
(DOCX)

S4 Table Appendage (spines and tube feet) lengths from sea urchins treated with DAPT and following regeneration over 29 days post amputation (dpa) (full time course experiment). Appendage length data means, ± s.e.m., n = 12 (full length spines), n = 6 (cut spines), n = 10–30 (tube feet, TF).
(DOCX)

S5 Table Appendage (spines and tube feet) lengths from sea urchins treated with DAPT and following regeneration over 29 days post amputation (dpa) (repeat experiment for gene expression data). Appendage length data are means, ± s.e.m., n = 12 (full length spines), n = 6 (cut spines), n = 10–30 (tube feet, TF).
(DOCX)
