# Supplementary material for: Tissue Regeneration and Biomineralization in Sea Urchins: Role of Notch Signaling and Presence of Stem Cell Markers
Source: PLoS One. 2015 Aug 12;10(8):e0133860. doi: 10.1371/journal.pone.0133860 (PMC4534296; doi:10.1371/journal.pone.0133860)
Supplement: S1 Fig — (DOCX) [file pone.0133860.s001.docx]

Pairwise Sequence Alignment > EMBOSS Needle

# vasa, isoform A [Drosophila melanogaster] GenBank: AAF53438 (amino acids 16-433)

# vasa [Lytechinus variegatus] GenBank: ACM80368

Lv_vasa 42 lrgg---------tsfekksyggsssgfgskang--------fg-igvgr

.||| .||..::.|....|.|.:..| || ||.||

Dm_vasaA 16 arggdwsddedtaksfsgeaegdgvggsggegggyqggnrdvfgrigggr

Lv_vasa 74 grgrgrgfqsfaeqggvggltngttngtsn--ggdsgwndppsngtsssp

|.|.| |::.....| ||...|...|..: ||:.|:. .|...|.

Dm_vasaA 66 gggag-gyrggnrdg--ggfhggrregerdfrggeggfr----ggqggsr

Lv_vasa 122 wddssssgagkrsfeskgsfgggggggrg------------gr----drg

.....|.| .:|.|.||.||.|| || :||

Dm_vasaA 109 ggqggsrg-------gqggfrggeggfrgrlyenedgderrgrldreerg

Lv_vasa 156 grsfg--dsenggggrsfxrpegeggeakppast----------------

|...| |.|..||.|. ..|:||.|:...:.

Dm_vasaA 152 gerrgrldreerggerg---ergdggfarrrrneddinnnnnivedverk

Lv_vasa 188 ---yippppseeeeqiyms-txqginfnryddipvevsgrdgpkhirsfe

||||.||.:..:|:.| . .||:|::|::|||:|:|.|.|:.|:.|.

Dm_vasaA 199 refyippepsndaieifssgiasgihfskynnipvkvtgsdvpqpiqhft

Lv_vasa 234 eagldetvlenvrkaryakptpvqkyaipiigagldlmacaqtgsgktaa

.|.|.:.:::||.|:.|..|||:||.:||:|.:|.|||||||||||||||

Dm_vasaA 249 sadlrdiiidnvnksgykiptpiqkcsipvissgrdlmacaqtgsgktaa

Lv_vasa 284 fllpiitnmitqsgcvscfsvvqeplalivsptrelasqiqnearkfcrn

|||||::.::.....:. :..|..:|||||||||.||.||||||...

Dm_vasaA 299 fllpilsklledphele----lgrpqvvivsptrelaiqifnearkfafe

Lv_vasa 334 tslrpvviyggtsvshqtrevqngcsilvatpgrmhdfigrgyiglgklk

:.|:..::|||||..||...:..||.:::|||||:.||:.|.:|.....:

Dm_vasaA 345 sylkigivyggtsfrhqnecitrgchvviatpgrlldfvdrtfitfedtr

Lv_vasa 384 ylildeadrmvdmgfgpeiqklidhphmppkgerqtlmfsatfppeiqek

:::|||||||:||||..::::::.|..|.| |.|||||||

Dm_vasaA 395 fvvldeadrmldmgfsedmrrimthvtmrp--ehqtlmfsa---------

Length: 450

Identity: 169/450 (37.6%)

Similarity: 232/450 (51.6%)

Gaps: 90/450 (20.0%)

Score: 687.5
