# Supplementary material for: Tissue Regeneration and Biomineralization in Sea Urchins: Role of Notch Signaling and Presence of Stem Cell Markers
Source: PLoS One. 2015 Aug 12;10(8):e0133860. doi: 10.1371/journal.pone.0133860 (PMC4534296; doi:10.1371/journal.pone.0133860)
Supplement: S1 Table — Annotated sequences for S. purpuratus were used to identify homologous sequences in the L. variegatus genome for primer design (www.echinobase.org). (DOCX) [file pone.0133860.s002.docx]

**S1 Table:** Gene-specific primers for *L. variegatus* for qRT-PCR analyses. Annotated sequences for *S. purpuratus* were used to identify homologous sequences in the *L. variegatus* genome for primer design (www.echinobase.org).

|  | **Gene identifier** | **Primer sequence (5’-3’)** | **Primer efficiency (*E*)** |
| --- | --- | --- | --- |
| **Notch target genes** | | |  |
| *hes* | SPU_021608 | F: CAACTACCACGCTGGCTTCA | 2.00 |
|  |  | Rv: TGTCGATGGTGTCGCAGTTC |  |
| *hey* | SPU_009465 | F: TGTCCTCTCACCCAATGGCTAT | 2.18 |
|  |  | Rv: ACCAGGGCAGTATGTGAGTTGA |  |
| *gataC* | SPU_027015 | F: CGGCTTATGCGTCGTTCAT  Rv: TCCCCATTACTCCTGGATGGT | 2.21 |
| *gcm* | SPU_006462 | F: CGCAGGACGTCCAAAGACA | 2.01 |
|  |  | Rv: TGCAGCATCGCTATCATGTACTG |  |
| **Stem cell markers** | |  |  |
| *vasa* | SPU_008908 | F: GGTGAGCGTCAGACCCTGAT | 2.03 |
|  |  | Rv: CGTTGAGGTACATCCTAGCCTTCT |  |
| *piwi* | SPU_023335 | F: TCCCGAGTTGTGCAGTCGTA | 2.03 |
|  |  | Rv: GCGTGGCTAGATCCTTCATGA |  |
| **Control genes** | | |  |
| *cyclophilin7* | SPU_008305 | F: CCTCCTTCCACAGGGTTATCC | 1.97 |
|  |  | Rv: GTACCGTTGCCCCTGGTAAA |  |
| *rpl8* | SPU_010692 | F: GCCAACAGGGCCATGGT | 2.02 |
|  |  | Rv: ACGCTTGACCTTGTATTTGAAGTAG |  |
| *profilin* | SPU_020197 | F: TGCAGGCGAGTAAGACAGCTATA | 1.98 |
|  |  | Rv: CTCCTTTATTCAAGTTCCCTTGCT |  |
| *ubiquitin* | SPU_021496 | F: CCATCACTCTTGAGGTTGAG | 1.96 |
|  |  | Rv: AGATGAGACGCTGCTGAT |  |
