# Supplementary material for: Tissue Regeneration and Biomineralization in Sea Urchins: Role of Notch Signaling and Presence of Stem Cell Markers
Source: PLoS One. 2015 Aug 12;10(8):e0133860. doi: 10.1371/journal.pone.0133860 (PMC4534296; doi:10.1371/journal.pone.0133860)
Supplement: S2 Table — Appendage length data are means, ± s.e.m., n = 12 (full length spines), n = 6 (regenerating spines), n = 10 (tube feet, TF). (DOCX) [file pone.0133860.s003.docx]

**S2 Table:** Appendage (spines and tube feet) lengths from sea urchins treated with vincristine and following regeneration over 29 days post amputation (dpa). Appendage length data are means, ± s.e.m., n=12 (full length spines), n=6 (regenerating spines), n=10 (tube feet, TF).

| **Animal #** | Vincristine (µg/g) | Sea urchin weight (g) | Sea urchin test diameter (mm) | Full length spines (mm) |  | | Appendage length (mm) | | | |
| --- | --- | --- | --- | --- | --- | --- | --- | --- | --- | --- |
|  |  |  |  |  |  | 8 dpa | | 15 dpa | 22 dpa | 29 dpa |
| 1 | 0 | 57.47 | 51.47 | 13.05 ± 0.15 | Regenerating spine | 4.34 ± 0.20 | | 5.41 ± 0.31 | 7.82 ± 0.28 | - |
|  |  |  |  |  | Full length TF | 17.43 ± 0.73 | | 17.56 ± 0.66 | 16.12 ± 0.41 |  |
|  |  |  |  |  | Regenerating TF | 5.01 ± 0.24 | | 8.42 ± 0.58 | 12.39 ± 0.39 |  |
| 2 | 0 | 56.11 | 51.28 | 12.59 ± 0.28 | Regenerating spine | 3.80 ± 0.15 | | 4.21 ± 0.20 | 7.01 ± 0.56 | 10.88 ± 0.43 |
|  |  |  |  |  | Full length TF | 15.36 ± 0.46 | | 16.33 ± 0.68 | 15.86 ± 0.48 | 12.72 ± 0.35 |
|  |  |  |  |  | Regenerating TF | 3.92 ± 0.27 | | 7.60 ± 0.49 | 10.13 ± 0.53 | 9.73 ± 0.87 |
| 3 | 0 | 45.86 | 50.01 | 11.87 ± 0.63 | Regenerating spine | 3.64 ± 0.27 | | 5.01 ± 0.29 | 8.22 ± 0.37 | 11.73 ± 0.36 |
|  |  |  |  |  | Full length TF | 18.57 ± 0.75 | | 15.63 ± 0.59 | 15.75 ± 0.63 | 17.25 ± 0.61 |
|  |  |  |  |  | Regenerating TF | 3.74 ± 0.18 | | 8.57 ± 0.51 | 11.42 ± 0.35 | 15.43 ± 0.62 |
| 4 | 0 | 34.21 | 43.13 | 11.68 ± 0.71 | Regenerating spine | 3.79 ± 0.15 | | 4.94 ± 0.26 | 7.53 ± 0.30 | 11.56 ± 0.36 |
|  |  |  |  |  | Full length TF | 17.95 ± 1.28 | | 15.88 ± 0.46 | 15.33 ± 0.40 | 15.94 ± 0.61 |
|  |  |  |  |  | Regenerating TF | 4.36 ± 0.34 | | 8.19 ± 0.57 | 10.96 ± 0.52 | 14.37 ± 0.64 |
| 5 | 0.2 | 57.43 | 53.74 | 11.80 ± 0.73 | Regenerating spine | 2.76 ± 0.17 | | 3.52 ± 0.19 | 5.14 ± 0.36 | 4.48 ± 0.40 |
|  |  |  |  |  | Full length TF | 17.87 ± 1.24 | | 16.70 ± 0.56 | 17.15 ± 0.43 | 16.31 ± 0.56 |
|  |  |  |  |  | Regenerating TF | 2.73 ± 0.34 | | 6.15 ± 0.58 | 8.52 ± 0.59 | 7.24 ± 0.33 |
| 6 | 0.2 | 43.09 | 46.45 | 11.49 ± 0.40 | Regenerating spine | 2.83 ± 0.19 | | 4.11 ± 0.22 | 5.11 ± 0.40 | 5.87 ± 0.37 |
|  |  |  |  |  | Full length TF | 15.55 ± 0.93 | | 14.99 ± 0.45 | 17.05 ± 1.01 | 16.37 ± 0.75 |
|  |  |  |  |  | Regenerating TF | 2.43 ± 0.15 | | 6.63 ± 0.49 | 9.98 ± 0.56 | 7.96 ± 0.31 |
| 7 | 0.2 | 26.89 | 41.89 | 11.73 ± 0.34 | Regenerating spine | 2.80 ± 0.18 | | 3.57 ± 0.24 | 5.19 ± 0.21 | 7.17 ± 0.37 |
|  |  |  |  |  | Full length TF | 15.45 ± 0.41 | | 15.89 ± 0.57 | 15.57 ± 0.60 | 16.68 ± 0.90 |
|  |  |  |  |  | Regenerating TF | 2.05 ± 0.17 | | 6.72 ± 0.51 | 7.71 ± 0.40 | 7.03 ± 0.54 |
| 8 | 0.2 | 34.93 | 44.0 | 10.74 ± 0.26 | Regenerating spine | 2.94 ± 0.08 | | 3.39 ± 0.25 | 4.02 ± 0.48 | 5.12 ± 0.37 |
|  |  |  |  |  | Full length TF | 18.01 ± 1.06 | | 15.28 ± 0.46 | 16.40 ± 0.66 | 16.14 ± 0.53 |
|  |  |  |  |  | Regenerating TF | 2.81 ± 0.40 | | 4.90 ± 0.57 | 8.23 ± 0.67 | 8.81 ± 0.54 |
| 9 | 0.6 | 51.41 | 49.09 | 10.96 ± 0.77 | Regenerating spine | 2.33 ± 0.16 | | - | - | - |
|  |  |  |  |  | Full length TF | 15.58 ± 0.53 | |  |  |  |
|  |  |  |  |  | Regenerating TF | 2.24 ± 0.16 | |  |  |  |
| 10 | 0.6 | 47.85 | 51.75 | 10.74 ± 0.45 | Regenerating spine | 2.43 ± 0.25 | | 2.16 ± 0.19 | - | - |
|  |  |  |  |  | Full length TF | 15.73 ± 0.54 | | 13.72 ± 0.70 |  |  |
|  |  |  |  |  | Regenerating TF | 1.95 ± 0.28 | | 2.42 ± 0.75 |  |  |
| 11 | 0.6 | 50.02 | 51.16 | 11.98 ± 0.53 | Regenerating spine | 2.01 ± 0.17 | | 2.13 ± 0.13 | 2.35 ± 0.31 | 3.32 ± 0.37 |
|  |  |  |  |  | Full length TF | 15.39 ± 0.52 | | 13.34 ± 0.55 | 15.22 ± 0.53 | 13.64 ± 0.58 |
|  |  |  |  |  | Regenerating TF | 3.85 ± 0.24 | | 3.16 ± 0.32 | 4.44 ± 0.27 | 3.76 ± 0.30 |
| 12 | 0.6 | 30.54 | 43.64 | 11.27 ± 0.46 | Regenerating spine | 1.98 ± 0.14 | | 2.24 ± 0.15 | 2.96 ± 0.19 | 3.82 ± 0.37 |
|  |  |  |  |  | Full length TF | 15.04 ± 0.60 | | 15.01 ± 0.65 | 14.71 ± 0.58 | 14.94 ± 0.48 |
|  |  |  |  |  | Regenerating TF | 2.65 ± 0.30 | | 4.29 ± 0.42 | 6.28 ± 0.38 | 5.85 ± 0.70 |
