# Supplementary material for: Tissue Regeneration and Biomineralization in Sea Urchins: Role of Notch Signaling and Presence of Stem Cell Markers
Source: PLoS One. 2015 Aug 12;10(8):e0133860. doi: 10.1371/journal.pone.0133860 (PMC4534296; doi:10.1371/journal.pone.0133860)
Supplement: S3 Table — Appendage length data are means, ± s.e.m., n = 18 (full length spines), n = 6 (cut spines), n = 10–30 (tube feet, TF). (DOCX) [file pone.0133860.s004.docx]

**S3 Table:** Appendage (spines and tube feet) lengths from sea urchins treated with DAPT and following regeneration over 15 days post amputation (dpa) (initial experiment). Appendage length data are means, ± s.e.m., n=18 (full length spines), n=6 (cut spines), n=10-30 (tube feet, TF).

| **Animal #** | DAPT (µg/g) | Full length spines (mm) |  | | Appendage length (mm) | |  |
| --- | --- | --- | --- | --- | --- | --- | --- |
|  |  |  |  |  | 8 dpa | 15 dpa | |
| 1 | 0 | 12.60 ± 0.23 | Regenerating spine | 2.07 ± 0.16 | | 4.70 ± 0.26 | |
|  |  |  | Full length TF | 18.28 ± 0.63 | | 18.47 ± 0.69 | |
|  |  |  | Regenerating TF | 4.69 ± 0.24 | | 11.05 ± 0.88 | |
| 2 | 0 | 14.78 ± 0.43 | Regenerating spine | 2.10 ± 0.18 | | 5.08 ± 0.23 | |
|  |  |  | Full length TF | 14.25 ± 0.53 | | 18.30 ± 0.61 | |
|  |  |  | Regenerating TF | 6.74 ± 0.44 | | 9.36 ± 0.60 | |
| 3 | 0 | 13.30 ± 0.58 | Regenerating spine | 2.28 ± 0.12 | | 4.85 ± 0.58 | |
|  |  |  | Full length TF | 16.36 ± 0.69 | | 18.36 ± 0.59 | |
|  |  |  | Regenerating TF | 5.19 ± 0.18 | | 11.31 ± 0.66 | |
| 4 | 0 | 16.12 ± 0.64 | Regenerating spine | 2.85 ± 0.23 | | 6.03 ± 0.16 | |
|  |  |  | Full length TF | 17.61 ± 0.57 | | 17.91 ± 0.49 | |
|  |  |  | Regenerating TF | 6.27 ± 0.48 | | 9.86 ± 1.07 | |
| 5 | 0.3 | 14.63 ± 0.74 | Regenerating spine | 2.48 ± 0.21 | | 5.52 ± 0.24 | |
|  |  |  | Full length TF | 16.78 ± 1.09 | | 17.63 ± 0.55 | |
|  |  |  | Regenerating TF | 4.08 ± 0.42 | | 10.66 ± 0.72 | |
| 6 | 0.3 | 15.17 ± 0.30 | Regenerating spine | 2.02 ± 0.20 | | 4.58 ± 0.35 | |
|  |  |  | Full length TF | 17.33 ± 0.62 | | 16.27 ± 0.91 | |
|  |  |  | Regenerating TF | 3.17 ± 0.15 | | 8.66 ± 0.53 | |
| 7 | 0.3 | 15.40 ± 0.36 | Regenerating spine | 2.53 ± 0.22 | | 5.17 ± 0.33 | |
|  |  |  | Full length TF | 17.04 ± 0.82 | | 17.78 ± 0.47 | |
|  |  |  | Regenerating TF | 4.25 ± 0.18 | | 6.59 ± 0.51 | |
| 8 | 0.3 | 15.83 ± 0.52 | Regenerating spine | 2.15 ± 0.19 | | 5.23 ± 0.37 | |
|  |  |  | Full length TF | 15.70 ± 0.89 | | 17.92 ± 0.56 | |
|  |  |  | Regenerating TF | 5.23 ± 0.16 | | 9.70 ± 0.54 | |
| 9 | 1 | 15.38 ± 0.43 | Regenerating spine | 2.20 ± 0.12 | | 5.25 ± 0.26 | |
|  |  |  | Full length TF | 15.83 ± 0.34 | | 18.62 ± 0.69 | |
|  |  |  | Regenerating TF | 3.74 ± 0.18 | | 8.82 ± 0.37 | |
| 10 | 1 | 15.92 ± 0.24 | Regenerating spine | 2.07 ± 0.22 | | 4.38 ± 0.25 | |
|  |  |  | Full length TF | 18.11 ± 0.75 | | 18.20 ± 0.70 | |
|  |  |  | Regenerating TF | 4.63 ± 0.17 | | 6.91 ± 0.32 | |
| 11 | 1 | 14.82 ± 0.59 | Regenerating spine | 2.35 ± 0.22 | | 5.25 ± 0.18 | |
|  |  |  | Full length TF | 18.79 ± 0.65 | | 18.81 ± 0.62 | |
|  |  |  | Regenerating TF | 3.73 ± 0.12 | | 6.31 ± 0.39 | |
| 12 | 1 | 11.80 ± 0.23 | Regenerating spine | 2.10 ± 0.13 | | 5.08 ± 0.10 | |
|  |  |  | Full length TF | 17.41 ± 0.74 | | 19.52 ± 0.69 | |
|  |  |  | Regenerating TF | 3.38 ± 0.19 | | 6.90 ± 0.30 | |
| 13 | 3 | 14.70 ± 0.34 | Regenerating spine | 1.92 ± 0.23 | | 4.95 ± 0.06 | |
|  |  |  | Full length TF | 14.77 ± 0.77 | | 20.15 ± 0.74 | |
|  |  |  | Regenerating TF | 3.51 ± 0.20 | | 5.86 ± 0.30 | |
| 14 | 3 | 18.07 ± 0.55 | Regenerating spine | 1.73 ± 0.16 | | 5.38 ± 0.22 | |
|  |  |  | Full length TF | 15.63 ± 0.60 | | 17.99 ± 0.47 | |
|  |  |  | Regenerating TF | 3.65 ± 0.18 | | 6.58 ± 0.16 | |
| 15 | 3 | 16.97 ± 0.39 | Regenerating spine | 2.12 ± 0.15 | | 5.13 ± 0.10 | |
|  |  |  | Full length TF | 17.00 ± 0.66 | | 18.31 ± 0.68 | |
|  |  |  | Regenerating TF | 4.60 ± 0.32 | | 7.18 ± 0.45 | |
| 16 | 3 | 16.8 ± 0.35 | Regenerating spine | 1.73 ± 0.13 | | 4.32 ± 0.32 | |
|  |  |  | Full length TF | 18.97 ± 0.75 | | 19.11 ± 0.85 | |
|  |  |  | Regenerating TF | 4.43 ± 0.42 | | 7.96 ± 0.25 | |
