# Supplementary material for: Tissue Regeneration and Biomineralization in Sea Urchins: Role of Notch Signaling and Presence of Stem Cell Markers
Source: PLoS One. 2015 Aug 12;10(8):e0133860. doi: 10.1371/journal.pone.0133860 (PMC4534296; doi:10.1371/journal.pone.0133860)
Supplement: S4 Table — Appendage length data means, ± s.e.m., n = 12 (full length spines), n = 6 (cut spines), n = 10–30 (tube feet, TF). (DOCX) [file pone.0133860.s005.docx]

**S4 Table:** Appendage (spines and tube feet) lengths from sea urchins treated with DAPT and following regeneration over 29 days post amputation (dpa) (full time course experiment). Appendage length data means, ± s.e.m., n=12 (full length spines), n=6 (cut spines), n=10-30 (tube feet, TF).

| **Animal #** | DAPT (µg/g) | Sea urchin weight (g) | Sea urchin test diameter (mm) | Full length spines (mm) |  | | Appendage length (mm) | | | | | | | | |
| --- | --- | --- | --- | --- | --- | --- | --- | --- | --- | --- | --- | --- | --- | --- | --- |
|  |  |  |  |  |  |  | 8 dpa | | 15 dpa | | 22 dpa | | 29 dpa | |  |
| 1 | 0 | 41.35 | 45.44 | 10.11 ± 0.26 | Regenerating spine | 2.92 ± 0.29 | | 3.37 ± 0.12 | | 5.29 ± 0.26 | | 6.38 ± 0.27 | |  |  |
|  |  |  |  |  | Full length TF | 43.90 ± 1.57 | | 30.97 ± 1.46 | | 36.38 ± 1.15 | | - | |  |  |
|  |  |  |  |  | Regenerating TF | 9.01 ± 0.20 | | 7.13 ± 0.50 | | 13.00 ± 1.13 | |  | |  |  |
| 2 | 0 | 38.38 | 41.76 | 9.75 ± 0.38 | Regenerating spine | 2.93 ± 0.29 | | 4.32 ± 0.44 | | 4.82 ± 0.32 | | 5.15 ± 0.45 | |  |  |
|  |  |  |  |  | Full length TF | 33.83 ± 0.57 | | 42.06 ± 1.09 | | 36.86 ± 1.00 | | 47.46 ± 1.16 | |  |  |
|  |  |  |  |  | Regenerating TF | 6.10 ± 0.46 | | 15.37 ± 0.92 | | 24.64 ± 1.60 | | 30.71 ± 1.66 | |  |  |
| 3 | 0 | 35.53 | 40.93 | 9.15 ± 0.32 | Regenerating spine | 2.83 ± 0.32 | | 3.67 ± 0.34 | | 5.05 ± 0.26 | | 6.04 ± 0.34 | |  |  |
|  |  |  |  |  | Full length TF | 35.71 ± 1.31 | | 39.13 ± 1.09 | | 28.16 ± 1.14 | | 44.48 ± 1.28 | |  |  |
|  |  |  |  |  | Regenerating TF | 6.79 ± 0.56 | | 14.15 ± 1.95 | | 15.74 ± 1.36 | | 24.42 ± 1.69 | |  |  |
| 4 | 0 | 27.15 | 36.34 | 10.03 ± 0.21 | Regenerating spine | 2.83 ± 0.20 | | 4.04 ± 0.29 | | 5.35 ± 0.44 | | 5.48 ± 0.25 | |  |  |
|  |  |  |  |  | Full length TF | 42.20 ± 2.06 | | 34.72 ± 1.17 | | 56.09 ± 0.93 | | 34.21 ± 1.18 | |  |  |
|  |  |  |  |  | Regenerating TF | 4.92 ± 0.40 | | 13.38 ± 1.43 | | 29.35 ± 2.36 | | 20.80 ± 1.75 | |  |  |
| 5 | 1 | 43.64 | 45.75 | 10.36 ± 0.25 | Regenerating spine | 3.32 ± 0.19 | | 3.92 ± 0.23 | | 3.79 ± 0.33 | | 4.61 ± 0.34 | |  |  |
|  |  |  |  |  | Full length TF | 37.37 ± 0.96 | | 42.26 ± 2.19 | | 34.89 ± 1.24 | | 54.24 ± 1.00 | |  |  |
|  |  |  |  |  | Regenerating TF | 5.36 ± 0.79 | | 10.27 ± 0.55 | | 15.32 ± 0.88 | | 30.19 ± 2.18 | |  |  |
| 6 | 1 | 39.71 | 41.23 | 10.79 ± 0.27 | Regenerating spine | 2.80 ± 0.28 | | 3.82 ± 0.10 | | 4.59 ± 0.40 | | 5.54 ± 0.58 | |  |  |
|  |  |  |  |  | Full length TF | 37.89 ± 1.28 | | 37.37 ± 0.76 | | 40.23 ± 1.75 | | 46.23 ± 1.43 | |  |  |
|  |  |  |  |  | Regenerating TF | 3.93 ± 0.38 | | 7.97 ± 0.67 | | 13.00 ± 1.00 | | 18.32 ± 1.30 | |  |  |
| 7 | 1 | 35.29 | 41.27 | 9.77 ± 0.30 | Regenerating spine | 2.94 ± 0.18 | | 4.13 ± 0.39 | | 3.93 ± 0.39 | | 5.52 ± 0.25 | |  |  |
|  |  |  |  |  | Full length TF | 32.94 ± 0.74 | | 32.50 ± 1.07 | | 41.99 ± 1.04 | | 41.01 ± 1.41 | |  |  |
|  |  |  |  |  | Regenerating TF | 3.92 ± 0.24 | | 9.25 ± 0.62 | | 21.70 ± 1.28 | | 15.47 ± 1.32 | |  |  |
| 8 | 1 | 22.98 | 36.49 | 10.40 ± 0.25 | Regenerating spine | 2.38 ± 0.36 | | 3.41 ± 0.21 | | 2.97 ± 0.24 | | 4.32 ± 0.28 | |  |  |
|  |  |  |  |  | Full length TF | 32.09 ± 072 | | 29.49 ± 0.87 | | 23.54 ± 0.81 | | 35.94 ± 0.66 | |  |  |
|  |  |  |  |  | Regenerating TF | 4.60 ± 0.17 | | 7.68 ± 0.28 | | 8.65 ± 0.59 | | 12.94 ± 0.76 | |  |  |
| 9 | 3 | 34.64 | 41.48 | 10.64 ± 0.28 | Regenerating spine | 2.80 ± 0.13 | | 3.41 ± 0.25 | | 4.14 ± 0.25 | | 4.69 ± 0.24 | |  |  |
|  |  |  |  |  | Full length TF | 28.88 ± 1.19 | | 31.35 ± 1.63 | | 29.23 ± 1.47 | | 37.79 ± 0.86 | |  |  |
|  |  |  |  |  | Regenerating TF | 2.74 ± 0.27 | | 7.69 ± 0.59 | | 8.77 ± 0.80 | | 11.38 ± 0.66 | |  |  |
| 10 | 3 | 44.52 | 43.49 | 10.73 ± 0.44 | Regenerating spine | 2.97 ± 0.19 | | 3.87 ± 0.47 | | 3.61 ± 0.40 | | 3.49 ± 0.26 | |  |  |
|  |  |  |  |  | Full length TF | 37.56 ± 0.94 | | 30.71 ± 0.99 | | 30.85 ± 0.98 | | 48.02 ± 2.36 | |  |  |
|  |  |  |  |  | Regenerating TF | 5.07 ± 0.46 | | 6.85 ± 0.73 | | 8.45 ± 0.58 | | 15.14 ± 0.95 | |  |  |
| 11 | 3 | 32.03 | 40.35 | 9.06 ± 0.20 | Regenerating spine | 2.72 ± 0.37 | | 3.11 ± 0.40 | | 3.48 ± 0.30 | | 3.51 ± 0.25 | |  |  |
|  |  |  |  |  | Full length TF | 42.52 ± 1.41 | | 42.60 ± 1.66 | | 41.56 ± 1.01 | | 54.34 ± 1.95 | |  |  |
|  |  |  |  |  | Regenerating TF | 5.88 ± 0.56 | | 8.60 ± 0.88 | | 9.61± 0.99 | | 15.18 ± 1.82 | |  |  |
| 12 | 3 | 27.88 | 38.28 | 9.90 ± 0.22 | Regenerating spine | 1.57 ± 0.12 | | 2.23 ± 0.22 | | 2.79 ± 0.33 | | 2.77 ± 0.34 | |  |  |
|  |  |  |  |  | Full length TF | 34.02 ± 1.01 | | 21.66 ± 0.88 | | 33.95 ± 0.51 | | 41.32 ± 0.98 | |  |  |
|  |  |  |  |  | Regenerating TF | 2.88 ± 0.22 | | 4.15 ± 0.56 | | 6.25 ± 0.73 | | 5.66 ± 0.27 | |  |  |
