# Supplementary material for: Tissue Regeneration and Biomineralization in Sea Urchins: Role of Notch Signaling and Presence of Stem Cell Markers
Source: PLoS One. 2015 Aug 12;10(8):e0133860. doi: 10.1371/journal.pone.0133860 (PMC4534296; doi:10.1371/journal.pone.0133860)
Supplement: S5 Table — Appendage length data are means, ± s.e.m., n = 12 (full length spines), n = 6 (cut spines), n = 10–30 (tube feet, TF). (DOCX) [file pone.0133860.s006.docx]

**S5 Table:** Appendage (spines and tube feet) lengths from sea urchins treated with DAPT and following regeneration over 29 days post amputation (dpa) (repeat experiment for gene expression data). Appendage length data are means, ± s.e.m., n=12 (full length spines), n=6 (cut spines), n=10-30 (tube feet, TF).

| **Animal #** | DAPT (µg/g) | Sea urchin weight (g) | Sea urchin test diameter (mm) | Full length spines (mm) |  | | Appendage length (mm) | | | | | | | |
| --- | --- | --- | --- | --- | --- | --- | --- | --- | --- | --- | --- | --- | --- | --- |
|  |  |  |  |  |  |  | 8 dpa | 15 dpa | | | 22 dpa | | 29 dpa | |
| 1 | 0 | 36.35 | 47 | 9.73 ± 0.41 | Regenerating spine | 3.17 ± 0.17 | | | | 7.16 ± 0.34 | | 8.60 ± 0.19 | | 8.74 ± 0.41 |
|  |  |  |  |  | Full length TF | 14.97 ± 0.57 | | | | 16.36 ± 0.60 | | 17.27 ± 1.11 | | 16.43 ± 1.23 |
|  |  |  |  |  | Regenerating TF | 3.84 ± 0.17 | | | | 8.89 ± 0.66 | | 13.75 ± 0.92 | | 16.27 ± 0.62 |
| 2 | 0 | 48.64 | 51 | 9.32 ± 0.47 | Regenerating spine | 3.12 ± 0.18 | | | | 6.32 ± 0.21 | | 6.93 ± 0.39 | | 8.26 ± 0.41 |
|  |  |  |  |  | Full length TF | 14.76 ± 0.82 | | | | 15.30 ± 0.73 | | 14.88 ± 0.57 | | 16.44 ± 0.67 |
|  |  |  |  |  | Regenerating TF | 3.84 ± 0.31 | | | | 8.46 ± 0.46 | | 13.54 ± 0.71 | | 14.44 ± 0.92 |
| 3 | 0 | 33.76 | 45 | 9.96 ± 0.51 | Regenerating spine | 3.20 ± .015 | | | | 5.85 ± 0.29 | | 7.84 ± 0.20 | | 8.94 ± 0.34 |
|  |  |  |  |  | Full length TF | 14.41 ± 0.82 | | | | 16.28 ± 0.78 | | 14.63 ± 0.56 | | 14.07 ± 0.61 |
|  |  |  |  |  | Regenerating TF | 3.53 ± 0.30 | | | | 8.32 ± 0.50 | | 12.08 ± 0.28 | | 13.83 ± 0.55 |
| 4 | 0 | 37.11 | 46 | 12.16 ± 0.46 | Regenerating spine | 3.48 ± 0.28 | | | | 6.64 ± 0.27 | | 7.23 ± 0.28 | | 9.27 ± 0.57 |
|  |  |  |  |  | Full length TF | 15.49 ± 0.99 | | | | 14.89 ± 0.76 | | 14.79 ± 0.49 | | 15.78 ± 0.67 |
|  |  |  |  |  | Regenerating TF | 3.89 ± 0.28 | | | | 8.99 ± 0.60 | | 13.27 ± 0.65 | | 15.40 ± 0.98 |
| 5 | 1 | 46.83 | 48 | 9.16 ± 0.43 | Regenerating spine | 3.02 ± 0.15 | | | | 6.24 ± 0.18 | | 7.17 ± 0.19 | | 9.08 ± 0.25 |
|  |  |  |  |  | Full length TF | 15.36 ± 0.76 | | | | 16.41 ± 0.55 | | 15.54 ± 0.74 | | 16.71 ± 0.78 |
|  |  |  |  |  | Regenerating TF | 3.62 ± 0.24 | | | | 8.61 ± 0.41 | | 12.63 ± 0.47 | | 13.59 ± 0.59 |
| 6 | 1 | 34.07 | 44 | 9.28 ± 0.47 | Regenerating spine | 3.05 ± 0.26 | | | | 6.08 ± 0.30 | | 8.16 ± 0.20 | | 8.00 ± 0.31 |
|  |  |  |  |  | Full length TF | 14.09 ± 0.85 | | | | 13.62 ± 0.57 | | 13.05 ± 0.71 | | 14.01 ± 0.29 |
|  |  |  |  |  | Regenerating TF | 4.24 ± 0.30 | | | | 7.76 ± 0.47 | | 9.40 ± 0.35 | | 12.31 ±0.73 |
| 7 | 1 | 36.19 | 44 | 9.37 ± 0.60 | Regenerating spine | 3.05 ± 0.16 | | | | 6.00 ± 0.60 | | 8.17 ± 0.27 | | 8.68 ± 0.38 |
|  |  |  |  |  | Full length TF | 16.00 ± 1.00 | | | | 15.67 ± 0.60 | | 16.03 ± 0.64 | | 16.33 ± 0.81 |
|  |  |  |  |  | Regenerating TF | 4.06 ± 0.26 | | | | 8.59 ± 0.52 | | 12.04 ± 1.01 | | 13.86 ± 0.70 |
| 8 | 1 | 38.08 | 48 | 8.81 ± 0.42 | Regenerating spine | 2.93 ± 0.17 | | | | 6.28 ± 0.24 | | 7.88 ± 0.26 | | 8.24 ± 0.19 |
|  |  |  |  |  | Full length TF | 16.44 ± 0.90 | | | | 15.48 ± 0.76 | | 16.42 ± 0.92 | | 15.98 ± 0.48 |
|  |  |  |  |  | Regenerating TF | 4.28 ± 0.42 | | | | 9.28 ± 0.52 | | 12.60 ± 0.97 | | 13.49 ± 0.63 |
| 9 | 3 | 39.68 | 44 | 10.10 ± 0.64 | Regenerating spine | 2.92 ± 0.18 | | | | 5.09 ± 0.34 | | 7.08 ± 0.30 | | 7.75 ± 0.19 |
|  |  |  |  |  | Full length TF | 16.34 ± 0.81 | | | | 16.02 ± 0.89 | | 14.70 ± 0.27 | | 16.05 ± 0.82 |
|  |  |  |  |  | Regenerating TF | 3.88 ± 0.30 | | | | 6.55 ± 0.65 | | 9.05 ± 0.65 | | 10.39 ± 0.71 |
| 10 | 3 | 46.69 | 49 | 10.90 ± 0.51 | Regenerating spine | 3.25 ± 0.30 | | | | 5.12 ± 0.25 | | 6.03 ± 0.47 | | 8.37 ± 0.30 |
|  |  |  |  |  | Full length TF | 14.87 ± 0.63 | | | | 16.41 ± 0.46 | | 14.44 ± 0.41 | | 14.89 ± 0.81 |
|  |  |  |  |  | Regenerating TF | 3.67 ± 0.37 | | | | 6.44 ± 0.42 | | 8.36 ± 0.51 | | 10.95 ± 0.77 |
| 11 | 3 | 34.25 | 47 | 10.63 ± 0.30 | Regenerating spine | 3.15 ± 0.19 | | | | 5.00 ± 0.33 | | 6.55 ± 0.18 | | 8.35 ± 0.28 |
|  |  |  |  |  | Full length TF | 13.97 ± 0.80 | | | | 16.81 ± 0.70 | | 14.83 ± 0.48 | | 14.35 ± 0.67 |
|  |  |  |  |  | Regenerating TF | 3.78 ± 0.42 | | | | 6.77 ± 0.36 | | 7.14 ± 0.33 | | 8.27 ± 0.41 |
| 12 | 3 | 31.77 | 46 | 11.05 ± 0.33 | Regenerating spine | 3.33 ± 0.09 | | | | 5.47 ± 0.20 | | 5.75 ± 0.20 | | 7.71 ± 0.28 |
|  |  |  |  |  | Full length TF | 14.09 ± 0.71 | | | | 15.52 ± 0.68 | | 15.92 ± 1.12 | | 15.71 ± 0.88 |
|  |  |  |  |  | Regenerating TF | 3.47 ± 0.26 | | | 6.91 ± 0.35 | | 9.05 ± 0.62 | | 10.28 ± 0.42 | |
| 13 | 9 | 44.70 | 46 | 10.75 ± 0.91 | Regenerating spine | 2.85 ± 0.22 | | | - | | - | | - | |
|  |  |  |  |  | Full length TF | 14.79 ± 0.62 | | |  | |  | |  | |
|  |  |  |  |  | Regenerating TF | 3.27 ± 0.20 | | |  | |  | |  | |
| 14 | 9 | 40.24 | 46 | 10.22 ± 0.98 | Regenerating spine | 3.35 ± 0.18 | | | - | | - | | - | |
|  |  |  |  |  | Full length TF | 15.58 ± 1.32 | | |  | |  | |  | |
|  |  |  |  |  | Regenerating TF | 3.44 ± 0.29 | | |  | |  | |  | |
| 15 | 9 | 33.34 | 46 | 9.67 ± 0.30 | Regenerating spine | 3.32 ± 0.15 | | | - | | - | | - | |
|  |  |  |  |  | Full length TF | 14.35 ± 0.77 | | |  | |  | |  | |
|  |  |  |  |  | Regenerating TF | 3.43 ± 0.41 | | |  | |  | |  | |
| 16 | 9 | 39.67 | 47 | 10.08 ± 0.53 | Regenerating spine | 3.12 ± 0.15 | | | - | | - | | - | |
|  |  |  |  |  | Full length TF | 14.43 ± 0.60 | | |  | |  | |  | |
|  |  |  |  |  | Regenerating TF | 3.89 ± 0.16 | | |  | |  | |  | |
